# The relationship between mental representations of self and social evaluation: Examining the validity and usefulness of visual proxies of self-image

**DOI:** 10.3389/fpsyg.2022.937905

**Published:** 2023-01-12

**Authors:** Jinwon Kim, Kibum Moon, Sojeong Kim, Hackjin Kim, Young-gun Ko

**Affiliations:** ^1^School of Psychology, Korea University, Seoul, South Korea; ^2^Department of Psychiatry, Korea University College of Medicine, Seoul, South Korea

**Keywords:** self-image, reverse correlation, visual representations, self-perception, self-evaluation, social evaluation

## Abstract

Reverse correlation (RC) method has been recently used to visualize mental representations of self. Previous studies have mainly examined the relationship between psychological aspects measured by self-reports and classification images of self (self-CIs), which are visual proxies of self-image generated through the RC method. In Experiment 1 (*N* = 118), to extend the validity of self-CIs, we employed social evaluation on top of self-reports as criterion variables and examined the relationship between self-CIs and social evaluation provided by clinical psychologists. Experiment 1 revealed that the valence ratings of self-CIs evaluated by independent raters predicted social evaluation after controlling for the effects of self-reported self-esteem and extraversion. Furthermore, in Experiment 2 (*N* = 127), we examined whether a computational scoring method – a method to assess self-CIs without employing independent raters – could be applied to evaluate the valence of participants’ self-CIs. Experiment 2 found that the computational scores of self-CIs were comparable to independent valence ratings of self-CIs. We provide evidence that self-CIs can add independent information to self-reports in predicting social evaluation. We also suggest that the computational scoring method can complement the independent rating process of self-CIs. Overall, our findings reveal that self-CIs are a valid and useful tool to examine self-image more profoundly.

## 1. Introduction

Self-image is an essential element of personality that has been studied for a long time (Rogers, 1959; Rosenberg, 1965; Coon, 1997). Self-image is defined as how we see ourselves, and it affects how we feel, think, and act in society (Rogers, 1959). Also, it is known as a multi-dimensional construct that includes subjective perceptions of not only oneself but also one’s own mental functioning, adjustment, and social attitudes in different areas of life (Lindfors et al., 2005; Di Blasi et al., 2015). Self-image is closely related to self-esteem (Rosenberg, 1965; Hulme et al., 2012), which can be viewed as an evaluative constituent of self-image (Lindfors et al., 2005). It is also linked to optimistic attitude toward life’s challenges (Mikulincer, 1995). In addition, negatively distorted self-image is a core feature of mental disorders, such as social anxiety disorder (Di Blasi et al., 2015; Meral and Vriends, 2022), body dysmorphic disorder (Didie et al., 2012), and eating disorder (Hrabosky et al., 2009). Furthermore, how people conceive themselves is associated with interpersonal relationships (Bartholomew and Horowitz, 1991; O’Koon, 1997) and behaviors in a social interaction situation (Hirsch et al., 2004).

Although self-image, or mental picture of self contains *imagery* properties (Bailey, 2003), traditional assessments of self-image have mainly employed verbal assessments (e.g., Offer et al., 1989; Amos et al., 1997; O’Koon, 1997). For example, Amos et al. (1997) found that participants with more positive self-image were more likely to relate themselves to positive adjectives such as “Healthy,” “Confident,” or “Nice” than to negative ones. In addition, Offer et al. (1989) developed the Offer Self-Image Questionnaire (QSIQ) to assess adolescents’ self-image. Recently, it has also been suggested that mental representations of self can be visualized by means of a technique called *reverse correlation* (Moon et al., 2020; Maister et al., 2021; Steiner et al., 2021). The reverse correlation (RC) method is a data-driven technique used to create a visualization of an individual’s mental representation (Dotsch et al., 2008, 2011; Brinkman et al., 2017; Brown-Iannuzzi et al., 2017). Application of the RC method in studying self-image allows researchers to investigate self-image in a novel way by visualizing mental representation of self (Moon et al., 2020; Maister et al., 2021; Steiner et al., 2021). In a RC image classification task designed to visualize an individual’s self-image, the individual selects the one out of a pair of faces that better resembles himself or herself across 300–500 trials. The presented facial stimuli consist of a single base face with superimposed random grayscale noise. By averaging the selected facial stimuli, one classification image of self (self-CI) is generated, which can be regarded as a visual proxy of mental representation of self (e.g., Moon et al., 2020).

Application of the RC method to measure self-image has notable advantages. The RC method incorporates participants’ spontaneous use of information to visualize their mental representations. In the RC task, participants freely adopt the criteria of their judgments that are necessary in selecting the stimuli (Brinkman et al., 2017). For example, some participants may choose stimuli that resemble themselves by focusing on facial features such as eyes, whereas others may select facial stimuli by focusing on more vague factors like overall impressions. Because participants can use criteria that come to mind without constraints when choosing facial stimuli, diverse criteria can be incorporated into the mental representation of self (Brinkman et al., 2017; Moon et al., 2020; Maister et al., 2021).

Another advantage of using the RC method is that researchers can visualize self-image with fewer biases due to social desirability. A typical RC paradigm uses a two-image forced choice RC task in which participants are forced to make spontaneous and instinctive decisions (Dotsch and Todorov, 2012). During the task, some participants may be unaware of the criteria that they adopt to select images (Brinkman et al., 2017). Therefore, mental representations visualized through the RC method may be less susceptible to social desirability as compared to explicit measures, such as self-reports (Pauzé et al., 2021). Supporting this argument, Moon et al. (2020) reported that participants’ social desirability was not significantly associated with their self-CIs but with self-reported variables related to self-image. This implies that application of the RC method in investigating self-image may allow us to further comprehend the features of self-image with fewer biases.

Prior studies have shown that the RC method can be a novel and promising method for studying self-image. In a pioneering study, Moon et al. (2020) provided evidence that the self-CIs generated through the RC method are valid proxies of mental representations of self. Participants reported that they perceived their self-CIs as bearing a stronger resemblance to themselves than did CIs of others, without knowing which images corresponded to their self-CIs. In addition, the valence ratings of self-CIs were significantly associated with self-image relevant variables (e.g., self-esteem, extraversion). Moreover, Steiner et al. (2021) utilized the RC technique to investigate the distortion and enhancement of one’s self-image in relation to narcissism. Their findings revealed that the narcissistic traits mediated the relationship between low self-concept clarity and self-image distortion, and that narcissistic insecurity mediated the relationship between the distortion of self-image and self-image enhancement. Furthermore, Maister et al. (2021) found that participants’ self-CIs were similar to their real faces, and that independent raters reliably inferred Big Five personality traits from self-CIs created by the RC method.

The existing studies have shown that the self-CIs generated by the RC method are related to self-reported psychological factors (e.g., Moon et al., 2020; Maister et al., 2021; Steiner et al., 2021). However, to the best of our knowledge, no empirical study has yet shown whether one’s self-CI would be associated with psychological aspects measured by methods other than self-reports. Therefore, to extend the validity of self-CIs, we employed social evaluation on top of self-reports as criterion variables and examined the relationship between self-CIs and social evaluation. Previous studies have constantly found that how an individual sees himself or herself may influence how that person is perceived by other people (Hirsch et al., 2004; Zeigler-Hill et al., 2013). For example, when the socially anxious people were asked to hold negative self-image in mind before a conversation with a stranger, they were evaluated more negatively by their partners in the quality of conversation than when they held a less negative self-image in mind (Hirsch et al., 2004). Similarly, Zeigler-Hill et al. (2013) reported that individuals with greater self-worth were evaluated more positively by others than were those with less self-worth. These studies may indicate that examining social evaluation contributes to a further understanding of self-image. In this respect, examining the relationship between social evaluation and self-CIs may provide additional evidence for the validity of the self-CIs.

In addition, given that the RC method incorporates the visual aspects, which is not included in self-reported measures (Moon et al., 2020), testing whether the self-CIs can provide incremental information to self-reports would help in investigating the usefulness of the RC method for studying self-image. Specifically, we aimed to examine the validity of self-CIs by investigating the association between the valence ratings of self-CIs and social evaluation provided by clinical experts (i.e., expert ratings). We also investigated the usefulness of the self-CIs by testing whether the self-CIs would provide incremental information in predicting social evaluation after controlling for the effects of self-reported measures.

Moreover, CIs have been mainly rated by independent raters on the judgments of interests (e.g., trustworthiness, dominance, and attractiveness; see Dotsch and Todorov, 2012; Brown-Iannuzzi et al., 2017). However, employing independent raters inevitably necessitates more time and effort. Therefore, we proposed a *computational scoring method* – a method to assess self-CIs more objectively and efficiently, eliminating the repeated process of recruiting independent raters to evaluate the self-CIs every time.

## 2. Experiment 1

The primary purpose of Experiment 1 was to examine the validity and usefulness of the self-CIs generated by the RC method, using both self-reports and expert ratings as the criterion variables. For this purpose, Experiment 1 consisted of three separate phases. In the first phase, participants completed self-reports, then had their facial photographs taken, and performed the RC task designed to generate their self-CIs. In addition, they evaluated whether their self-CIs resembled themselves. Also, they recorded a 5-min self-introduction video for social evaluation. In the second phase, we recruited a new sample of independent raters. The independent raters evaluated the valence of the participants’ self-CIs and facial appearance. In the final phase, licensed clinical psychologists evaluated the psychological adjustment of participants based on the self-introduction videos.

We hypothesized that participants with higher valence ratings of self-CIs would be rated more positively by clinical experts in terms of psychological adjustment than would be those with lower valence ratings of self-CIs. Moreover, we postulated that the valence ratings of self-CIs would predict social evaluations provided by experts even after controlling for the effects of self-reported features related to self-image and facial appearance. In addition to main hypothesis, we postulated that the valence ratings of self-CIs would be positively correlated with self-reported self-esteem, extraversion, and explicit self-evaluation. Lastly, we expected that the valence ratings of self-CIs would not be significantly associated with social desirability.

### 2.1. Methods

#### 2.1.1. Participants

In Experiment 1, we recruited 118 undergraduate students (87 females and 31 males) to perform the RC task *via* an online advertisement and printed flyers. The mean age of participants was 20.92 (*SD_age_* = 2.02; age range = 18–27). They received a $15 gift voucher for their participation. They signed a written informed consent form. Additionally, we recruited 59 independent raters (29 females and 30 males; *M_age_* = 23.00, *SD_age_* = 2.88; age range = 19–34) to evaluate the valence of the participants’ self-CIs and facial appearance. The independent raters consisted of 49 undergraduate students (83.05%) and 10 graduate students (16.95%). They received $25 for their participation. Before evaluating the images, they signed a consent form. All participants and independent raters were Asian.

To blind participants to our hypothesis, before the experiment we said that we were examining the relationship between personality traits and the ways that people perceive social stimuli. We debriefed the purpose of this study after the experiment. Experiment 1 was approved by the Institutional Review Board.

#### 2.1.2. Materials and procedures

##### 2.1.2.1. Self-reports

###### 2.1.2.1.1. Rosenberg self-esteem scale (RSES)

We used the RSES, a 10-item measure originally developed by Rosenberg (1965) and later validated in Korean (Lee and Won, 1995), to assess global self-esteem. Each question is answered on a 5-point Likert scale (1 = *not very true of me*; 5 = *very true of me*). The internal consistency of the RSES was 0.86.

###### 2.1.2.1.2. Explicit self-evaluation

Participants rated how they evaluated themselves using seven items from 14 self-presentation domains (Leary and Allen, 2011). Each question was scored on a 9-point bipolar scale (e.g., unfriendly, unlikable vs. friendly, likable). We used the Korean version of explicit self-evaluation (Moon et al., 2020). The internal consistency of explicit evaluation was 0.65.

###### 2.1.2.1.3. Extraversion

We used 10 items related to extraversion in the HEXACO-60 scale to assess extraversion (Ashton and Lee, 2009).^1^ The HEXACO-60 is answered on a 5-point Likert scale (1 = *strongly disagree*; 5 = *strongly agree*). The extraversion in the HEXACO-60 includes the factors of social self-esteem, social boldness, sociability, and liveliness. We used the Korean version of HEXACO-60 (Lee and Ashton, 2013). The internal consistency was 0.77.

###### 2.1.2.1.4. Center for epidemiological studies depression scale (CES-D)

We used the CES-D, a 20-item measure developed by Randloff (1977) and later validated in Korean (Chon et al., 2001), to assess depressive symptoms. Each question ranged from “0 = rarely or none of the time (less than 1 day per week)” to “3 = most or all the time (5 to 7 days in a week).” The internal consistency of the CES-D was 0.91.

###### 2.1.2.1.5. Taylor manifest anxiety scale (TMAS)

We used the TMAS, developed by Bendig (1956) and later validated in Korean (Lee, 2000a), to assess chronic anxiety symptoms. The TMAS consisted of 20 binary items (Cronbach’s α = 0.84).

###### 2.1.2.1.6. Marlowe-Crowne social desirability scale (MCSDS)

We used the MCSDS, originally developed by (Crowne and Marlowe, 1960) and later validated in Korean (Lee, 2000b), to measure social desirability. This scale consists of 33 binary items (Cronbach’s α = 0.77).

##### 2.1.2.2. Facial photographs

After completing self-reported questionnaires, participants had their photographs taken. To control for the effect of extraneous factors on the evaluation of the facial photographs, we asked participants to (1) put on neutral facial expression, (2) take off all accessories, including glasses and visible jewelry, and (3) tie their hair back to show ears if necessary. We then cropped the facial photographs from the top of the head to the neck and aligned the photographs so that every facial feature is in the same position in every photograph by using Python and the OpenCV library (Bradski, 2000). In addition, we converted all facial photographs into black and white, since CIs were in black and white.

##### 2.1.2.3. Reverse correlation (RC) task

**Figure 1:**
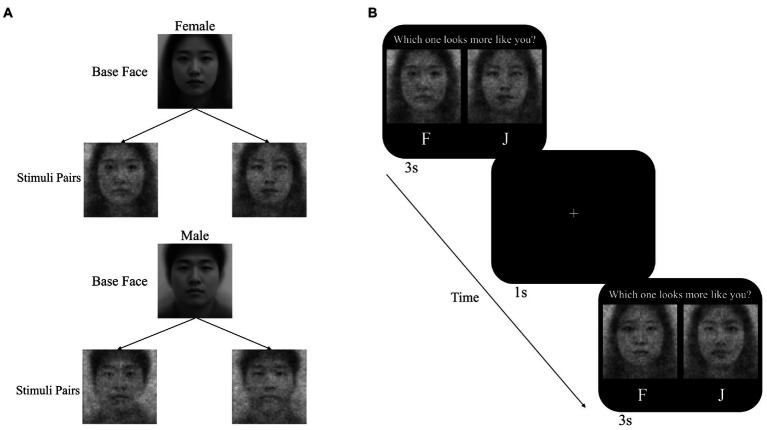
**(A)** Base faces and examples of stimuli pairs used in the reverse correlation task. **(B)** Illustrations of the image-generation phase.

Participants then performed the RC task to generate their self-CIs. Facial stimuli used in the RC task were generated from two base faces, which are morphed composites of 100 Asian faces for each sex (Moon et al., 2020). Using the rcicr package (Dotsch, 2016), we superimposed random grayscale noise on each base face to generate 300 pairs of facial stimuli per sex. Each pair of stimuli included a particular noise pattern and its inverse noise pattern (see Figure 1A). The inverse noise pattern is the mathematical opposite of the particular noise pattern, which makes facial stimuli look different with each noise pattern (Dotsch and Todorov, 2012).

Participants completed 300 trials of the RC task to select an image that bore a stronger resemblance to themselves from a pair of images to each generate a self-CI (see Figure 1B). On each trial, two facial stimuli were presented side by side. Upon presenting a pair of stimuli, participants were forced to choose the one from two facial stimuli within 3 s (Moon et al., 2020). The 300 pairs of facial stimuli were presented in random order. Using the rcicr package (Dotsch, 2016), we generated a self-CI for each participant by superimposing the averaged noise of all selected images on the base face. The R codes found in the repository^2^ provide a tutorial for generating self-CIs. We computerized the entire procedure of the RC task using the PsychoPy program (Peirce et al., 2019).

##### 2.1.2.4. Resemblance ratings of self-CIs

Upon the completion of the RC task, participants evaluated their self-CIs on resemblance without knowing that the self-CIs were generated from 300 trials of the RC task.^3^ The purpose of the resemblance evaluation was to test whether the self-CIs of participants reflected their facial appearance as part of a manipulation check. As Moon et al. (2020) did, we included five filler-CIs per sex with their self-CIs to check whether participants perceived their self-CIs as more similar to themselves than were the filler-CIs. We utilized the filler-CIs used in Moon et al. (2020). The six CIs (a participant’s self-CI and five filler-CIs) were presented in random to avoid experimental biases. The resemblance was rated on a 9-point Likert scale (1 = *weaker resemblance to myself*; 9 = *stronger resemblance to myself*).

##### 2.1.2.5. Videotaped self-introductions

Participants were asked to freely introduce themselves to potential job interviewers for 5-min as part of a job interview simulation. Before the self-introduction task, we set the laptop camera to match the eye-level of the participants to capture non-verbal communication (e.g., gestures, facial expression, and eye contact). Participants were informed that their brief videos were evaluated by three clinical psychologists.

##### 2.1.2.6. Independent valence ratings of self-CIs and facial appearance

In the second phase, the independent raters evaluated both the valence of the participants’ facial appearance and self-CIs without knowing the study hypotheses or how the self-CIs were generated. They performed the evaluation tasks on an online experimental platform^4^. We randomly assigned the raters to two groups, considering the sex ratio and the fact that evaluating too many images might cause fatigue and reduce the reliability of the evaluation. Two groups of randomly assigned independent raters evaluated 59 self-CIs of the participants and the same number of facial photographs on the valence.

We used the seven items from 14 self-presentational domains (Leary and Allen, 2011) to assess the valence of self-CIs and facial appearance (see Moon et al., 2020 for specific items). The items were rated on 9-point bipolar scale (e.g., 1 = “unfriendly, unlikable,” 9 = “friendly, likable”). We averaged the result of the seven items to calculate one independent valence rating. All items were presented in random order. The independent raters evaluated the next image after all seven items were evaluated for one image. The internal consistency of the independent valence ratings of self-CIs and facial appearance was 0.97 and 0.92, respectively.

##### 2.1.2.7. Evaluations of self-introduction videos

In the final phase, three licensed clinical psychologists (1 female and 2 males) completed evaluations of the participants’ psychological adjustment level based on their self-introduction videos. Three clinical experts were unaware of the study hypotheses. The perceived psychological adjustment was measured with five items on 9-point Likert scales: (1) emotional instability, (2) psychological maturity, (3) interpersonal competence, (4) psychological flexibility, and (5) invoking positive emotions.^5^ These five items were averaged to calculate one expert rating (α = 0.98). Inter-rater reliability of expert ratings proved to be good using intraclass correlation coefficients (ICC; Cicchetti, 1994) for the averaged expert ratings of ICC(2, k) = 0.62, *p* < 0.001, and ICC(3, k) = 0.70, *p* < 0.001.

### 2.2. Results and discussion

#### 2.2.1. Resemblance rating

We examined whether participants perceived that their self-CIs reflected their facial appearances as part of a manipulation check. A paired sample t-test revealed that participants perceived their self-CIs (*M* = 5.63, *SD* = 1.89, 95% CI [5.28, 5.97]) to be more similar to themselves than were filler-CIs (*M* = 4.02, *SD* = 0.99, 95% CI [3.84, 4.20]) in the resemblance rating, *t*(117) = 8.47, *p* < 0.001.

#### 2.2.2. Relationship between independent valence ratings of self-CIs and self-reported variables

As shown in Table 1, valance ratings of self-CIs rated by independent raters were positively correlated with self-esteem (*r* = 0.23, *p* < 0.05), extraversion (*r* = 0.29, *p* < 0.01), and explicit self-evaluation (*r* = 0.29, *p* < 0.01). The independent valence ratings of self-CIs were negatively correlated with trait anxiety (*r* = −0.24, *p* < 0.05), and were not significantly correlated with depression (*r* = −0.11, *p* = 0.222) or social desirability (*r* = 0.18, *p* = 0.054). This is consistent with the findings of Moon et al. (2020) that the self-CIs are associated with one’s attitude toward oneself and personality traits related to interpersonal relationships.

**Table 1:** Descriptive statistics and correlations between study variables.

	1	2	3	4	5	6	7	8	9
1. VR_IR_	–								
2. VR_FA_	0.37***	–							
3. Expert ratings	0.28**	0.12	–						
4. Self-esteem	0.23*	0.19*	0.21*	–					
5. Explicit self-evaluation	0.29**	0.24**	0.16	0.49***	–				
6. Extraversion	0.29**	0.16	0.29**	0.63***	0.52***	–			
7. Depression	−0.11	−0.14	−0.08	−0.67***	−0.37***	−0.48***	–		
8. Anxiety	−0.24*	−0.22*	−0.15	−0.64***	−0.35***	−0.49***	0.70***	–	
9. Social desirability	0.18	0.09	−0.06	0.19*	0.23*	0.08	−0.21*	−0.28**	–
*M*	4.76	5.18	5.34	28.81	6.45	30.76	16.56	8.78	16.42
*SD*	0.86	0.59	1.20	5.55	0.79	5.95	9.99	4.67	5.13

*N* = 118, VR_IR_ = Valence ratings of the independent raters (self-CIs); VR_FA_ = Valence ratings of facial appearance. *p < 0.05, **p < 0.01, ***p < 0.001.

#### 2.2.3. Relationship between independent valence ratings of self-CIs and facial appearance

Because the correlation between independent valence ratings of self-CIs and facial appearance was significant (*r* = 0.37, *p* < 0.001), we conducted a multiple linear regression analysis to examine whether the mental representation of self is predicted by psychological factors even after controlling for actual facial appearance. Specifically, we entered both self-reported explicit self-evaluation and independent valence ratings of facial appearance as predictor variables, and independent valence ratings of self-CIs as a dependent variable in the model. We included explicit self-evaluation among self-reported variables, because explicit self-evaluation was measured with the same items used in the independent valence ratings. Explicit self-evaluation predicted the independent valence ratings of self-CIs, β = 0.21, *t*(115) = 2.46, *p* < 0.05, 95% CI [0.04, 0.39], even after controlling for the independent valence ratings of facial appearance, β = 0.32, *t*(115) = 3.66, *p* < 0.001, 95% CI [0.15, 0.49]. In line with Maister et al. (2021), we provide evidence that individuals’ self-CIs do not simply reflect their facial appearances but are influenced by psychological factors.

#### 2.2.4. Relationship between independent valence ratings of self-CIs and expert ratings

To examine the validity and usefulness of the self-CIs generated by the RC method, we investigated the relationship between the independent valence ratings of self-CIs and expert ratings on psychological adjustment after watching participants’ self-introductory videos. As presented in Table 1, we found that expert ratings on psychological adjustment were significantly correlated with the independent valence ratings of self-CIs (*r* = 0.28, *p* < 0.01) but not with the independent valence ratings of facial appearance (*r* = 0.12, *p* = 0.212). Among self-reported variables related to self-image, extraversion was significantly correlated with expert ratings on psychological adjustment (*r* = 0.29, *p* < 0.01), as was self-esteem (*r* = 0.21, *p* < 0.05). On the other hand, other self-reported variables were not significantly correlated with psychological adjustment (|*r*|s = 0.06 ~ 0.16, *p* = ns).

Given that extraversion and self-esteem were positively correlated with the psychological adjustment as evaluated by experts, we performed a multiple linear regression analysis to investigate the independent and incremental effect of the valence of self-CIs on expert ratings after controlling for the effects of extraversion and self-esteem. The relationship between the independent valence ratings of self-CIs and expert ratings remained significant, β = 0.21, *t*(114) = 2.32, *p* < 0.05, 95% CI [0.03, 0.39], after controlling for the effects of extraversion, β = 0.20, *t*(114) = 1.77, *p* = 0.080, 95% CI [−0.02, 0.43], and self-esteem, β = 0.03, *t*(114) = 0.30, *p* = 0.762, 95% CI [−0.19, 0.26].

In support of our main hypothesis, our findings revealed that higher the independent valence ratings of self-CIs, participants were evaluated more positively by experts. This is consistent with previous literature that individuals with positive self-perceptions are viewed more favorably by others than those with negative self-perceptions (Taylor et al., 2003; Zeigler-Hill et al., 2013). Building on previous studies, we demonstrated that the significant relationship between self-image and social evaluation can be revealed through the RC method. In addition, these findings imply that the self-CIs add information to self-reported variables in predicting social evaluation. This suggests that the RC method can be a valid and useful tool for understanding the features of self-image that are hard to capture with self-reports.

## 3. Experiment 2a

The independent-rating method necessitates a considerable number of independent raters to reliably evaluate participants’ self-CIs generated through the RC method. In addition, the independent-rating method requires an additional recruitment of raters each time participants’ self-CIs are evaluated. In Experiment 2a, we aimed to propose a computational scoring method to efficiently and objectively measure self-CIs as an alternative approach to independent ratings.

### 3.1. Methods

#### 3.1.1. Participants

Participants in Experiment 1 visited the laboratory again about a month after the entire completion of Experiment 1. Experiment 2a was also approved by the Institutional Review Board.

#### 3.1.2. Materials and procedures

##### 3.1.2.1. Computational scoring method

**Figure 2:**
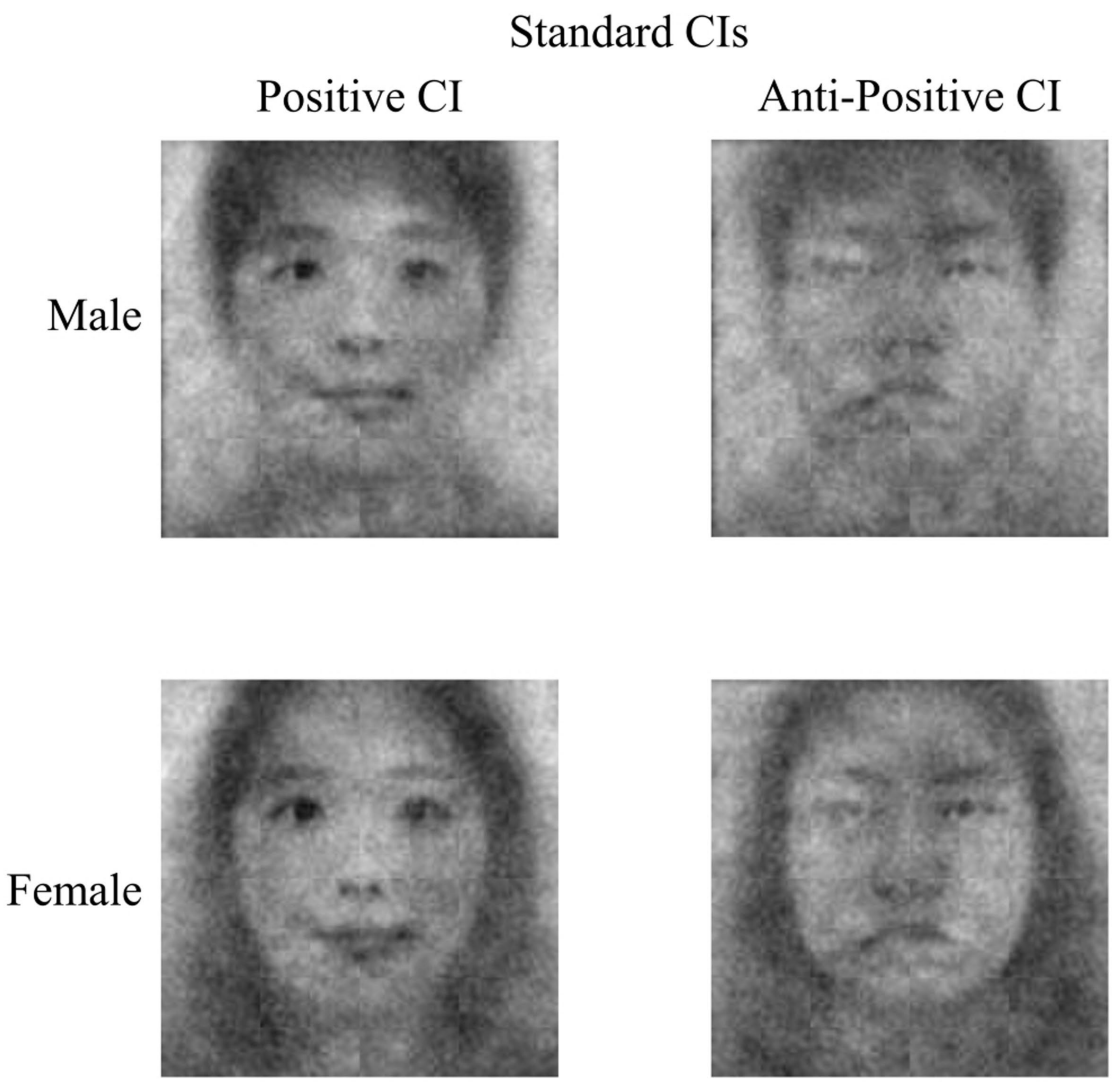
The standard classification images (CIs) generated by superimposing the averaged grayscale noise patterns of all selected images on the base images (left) and all non-selected images on the base images (right).

To compute the valence of self-CIs objectively and efficiently, the RC task was designed to select an image that looked more positive, with the instruction, “Which one looks more positive?” The instruction was adapted from a study by Dotsch and Todorov (2012). Based on the number of times a positive stimulus was selected from each pair, the positivity score of all stimuli used in the RC task was computed. For example, if 40 percent of participants chose the image on the left in a certain pair, the left image was granted a positivity score of 0.4, while the score of the right image was coded as 0.6. Given that each facial stimulus has a positivity score, the total positivity score, which we refer to as *computational scores* can be calculated by averaging all positivity scores for the images selected to generate self-CIs. For example, if a participant selected images with positivity scores of 0.4, 0.5, and 0.6, the total positivity score was 0.5. Repeating this method, the total positivity score can be computed for 300 selected images (see Figure 2 for procedure details). Considering the occurrence of non-response caused by the 3 s limit for each trial, the positivity scores of the participants’ self-CIs generated in Experiment 1 were not summed but averaged. The R codes found in the repository^6^ provide the method of granting the positivity scores of all facial stimuli presented in the RC task and calculating the positivity scores of self-CIs.

##### 3.1.2.2. Independent valence ratings of positive-CIs

**Figure 3:**
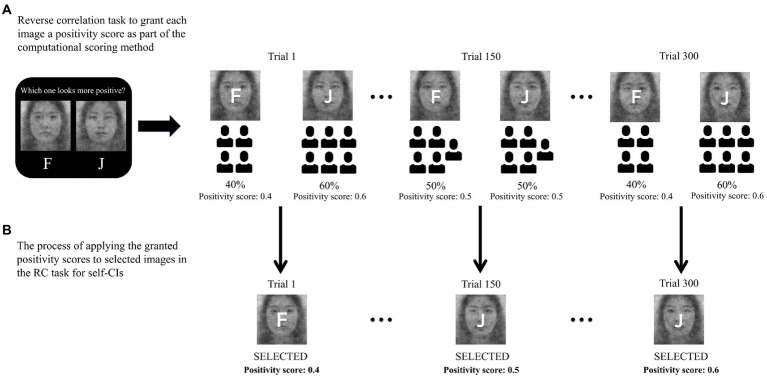
Procedure to evaluate positivity of the selected images in the reverse correlation (RC) task for self-CIs, which we named the computational scoring method. **(A)** RC task was performed to select the more positive image out of two to grant each image a positivity score as part of the computational scoring method. **(B)** The granted positivity score in **(A)** was applied to the selected images in the RC task for self-CIs, in which participants chose one that bore stronger resemblance.

To test whether participants reliably selected facial stimuli that looked more positive, the aforementioned independent raters evaluated the valence of the positive-CIs, each created by the participants with the same items as those used to assess the valence of self-CIs in Experiment 1. To be specific, we conducted a paired sample t-test to examine whether the positive-CIs created in Experiment 2a were evaluated more positively than the self-CIs of participants. We found that the independent raters perceived the positive-CIs created by the participants (*M* = 5.34, *SD* = 0.58, 95% CI [5.23, 5.44]) as more positive than the participants’ self-CIs (*M* = 4.76, *SD* = 0.86, 95% CI [4.60, 4.92]), *t*(117) = 6.01, *p* < 0.001. For the descriptive purpose, we superimposed the averaged grayscale visual noise of all selected images on the base images to create standard positive-CIs by sex. In an equivalent manner, we created standard anti-positive CIs for all non-selected images (see Figure 3).

### 3.2. Results and discussion

#### 3.2.1. Validity of the computational scores of self-CIs

The validity of computational scores (positivity scores) of self-CIs created in Experiment 1 was examined by assessing their relationship with variables used in Experiment 1 (self-reported variables and social evaluations by experts). In other words, we tested whether computational scores of self-CIs can substitute independent ratings of self-CIs. We found that the computational scores of self-CIs were strongly correlated with the independent valence ratings of self-CIs (*r* = 0.86, *p* < 0.001). In addition, the computational scores of self-CIs were significantly correlated with self-esteem (*r* = 0.22, *p* < 0.05), explicit self-evaluation (*r* = 0.32, *p* < 0.001), extraversion (*r* = 0.33, *p* < 0.001), trait anxiety (*r* = −0.23, *p* < 0.05), and psychological adjustment as evaluated by experts (*r* = 0.33, *p* < 0.001).

Moreover, we reanalyzed the multiple linear regression model predicting participants’ social evaluations provided by the clinical experts. We entered the computational scores of self-CIs as a predictor variable instead of the independent valence ratings of self-CIs. We found that the effect of the computational scores of self-CIs on expert ratings remained significant, β = 0.26, *t*(114) = 2.86, *p* < 0.01, 95% CI [0.08, 0.44], even after controlling for the effect of extraversion, β = 0.17, *t*(114) = 1.50, *p* = 0.136, 95% CI [−0.06, 0.40] and self-esteem, β = 0.04, *t*(114) = 0.40, *p* = 0.694, 95% CI [−0.18, 0.27]. Overall, these findings revealed that the computational scoring method could be a valid approach in assessing the self-CIs and may supplement the commonly used independent rating method.

## 4. Experiment 2b

To examine whether the computational scoring method made in Experiment 2a is valid and applicable to evaluate newly recruited participants’ self-CIs, we replicated the findings of Experiment 2a. For this purpose, Experiment 2b comprised two separate phases. In the first phase, newly recruited participants answered a set of self-reported measures. They then performed the RC task to generate their self-CIs and evaluated these images in terms of resemblance. Because the entire process was conducted online, participants were guided to perform the RC task and the resemblance evaluation in a quiet environment as much as possible. We calculated the computational scores of the participants’ self-CIs upon the completion of task. In the second phase, a new sample of independent raters evaluated the valence of the participants’ self-CIs.

We hypothesized that the computational scores of self-CIs would be correlated with the valance ratings of self-CIs as evaluated by independent raters and self-reported variables related to self-image, such as self-esteem, explicit self-evaluation, and extraversion.

### 4.1. Methods

#### 4.1.1. Participants

In Experiment 2b, we recruited 127 participants (86 females and 41 males) that performed the RC task *via* an online advertisement and printed flyers. They consisted of 91 undergraduate students (71.65%) and 36 graduate students (28.35%). The mean age of participants was 25.35 (*SD_age_* = 5.41; age range = 18–47). For their participation, they received a $10 gift voucher. They voluntarily signed a written informed consent form. In addition to the participants, we recruited 62 independent raters (32 females and 30 males) to rate the valence of self-CIs generated by the participants. The independent raters consisted of 53 undergraduate students (85.48%) and 9 graduate students (14.52%). The mean age of the independent raters was 22.45 (*SD_age_* = 2.47; age range = 19–31). They received $10 for their participation. They provided informed consent electronically. All participants and independent raters were Asian. Experiment 2b was also approved by the Institutional Review Board.

#### 4.1.2. Materials and procedures

##### 4.1.2.1. Self-reports

We used the questionnaires used in Experiment 1. The internal consistency of each self-reported measure is as follows: RSES, α = 0.92, Explicit Self-Evaluation, α = 0.73; Extraversion, α = 0.86; CES-D, α = 0.93; TMAS, α = 0.89; MCSDS, α = 0.64.

##### 4.1.2.2. Reverse correlation (RC) task

For the RC task, we utilized 300 pairs of facial stimuli used in Experiment 1. Unlike Experiment 1, the RC task was performed *via* an online experimental platform (pavlovia.org). The remaining detailed procedures of the RC task were identical to those in Experiment 1 (see Figure 1B).

##### 4.1.2.3. Resemblance ratings of self-CIs

Participants evaluated how similar their self-CIs were to themselves online 1 week after the completion of the RC task.

##### 4.1.2.4. Independent valence ratings of self-CIs

One group of independent raters (32 raters) evaluated 64 of the 127 CIs, while the other group (30 raters) evaluated the rest. As did in Experiment 1, the independent raters evaluated the valence of the participants’ self-CIs with the seven items *via* the online platform. The internal consistency of the independent ratings of self-CIs was 0.97.

### 4.2. Results and discussion

#### 4.2.1. Resemblance rating

A paired sample t-test revealed that participants perceived their self-CIs (*M* = 5.53, *SD* = 1.86, 95% CI [5.20, 5.85]) as bearing a stronger resemblance to themselves than filler-CIs (*M* = 3.71, *SD* = 1.04, 95% CI [3.53, 3.89]) in the resemblance ratings, *t*(126) = 10.14, *p* < 0.001.

#### 4.2.2. Relationship between independent valence ratings of self-CIs and self-reported variables

All the significant correlations between the independent valence ratings of self-CIs and variables related to self-image in Experiment 1 were replicated in Experiment 2b. To be specific, as presented in Table 2, the valence ratings of self-CIs evaluated by independent raters were significantly associated with self-esteem (*r* = 0.19, *p* < 0.05), explicit self-evaluation (*r* = 0.32, *p* < 0.001), and extraversion (*r* = 0.31, *p* < 0.001). In Experiment 2b, trait anxiety was not significantly correlated with the independent valence ratings of self-CIs (*r* = −0.09, *p* = 0.313). Meanwhile, the valence ratings of independent raters did not show significant correlations with depression symptoms (*r* = −0.15, *p* = 0.098) or social desirability (*r* = −0.03, *p* = 0.769).

**Table 2:** Descriptive statistics and correlations between study variables.

	1	2	3	4	5	6	7	8
1. VR_IR_	–							
2. CS	0.80***	–						
3. Self-esteem	0.19*	0.25**	–					
4. Explicit self-evaluation	0.32***	0.38***	0.71***	–				
5. Extraversion	0.31***	0.37***	0.77***	0.71***	–			
6. Depression	−0.15	−0.20*	−0.80***	−0.55***	−0.59***	–		
7. Anxiety	−0.09	−0.20*	−0.78***	−0.51***	−0.63***	0.73***	–	
8. Social desirability	−0.03	0.05	0.34***	0.28**	0.17	−0.25***	−0.34***	–
*M*	4.90	0.54	28.61	6.46	30.98	19.09	9.72	16.28
*SD*	0.83	0.04	6.66	1.00	7.09	11.78	5.36	4.17

*N* = 127, VR_IR_ = Valence ratings of the independent raters (self-CIs); CS = Computational scores (self-CIs). *p < 0.05, **p < 0.01, ***p < 0.001.

#### 4.2.3. Replication of Experiment 2a: Validity of the computational scores of self-CIs

**Figure 4:**
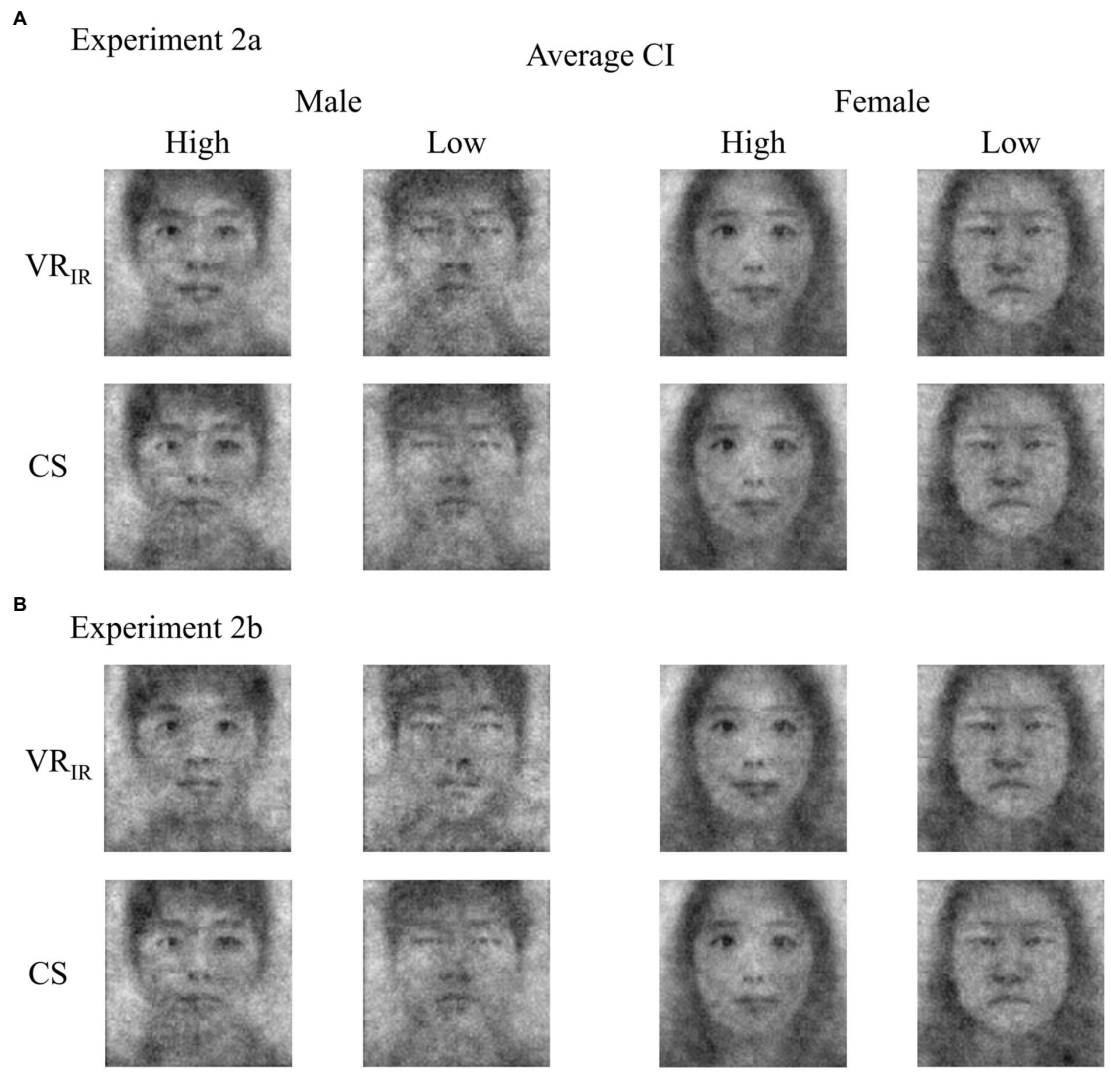
The average classification images of self (self-CIs) generated in Experiment 2a **(A)** and Experiment 2b **(B)** by high (+1 *SD*) and low (−1 *SD*) groups of independent valence ratings and of computational scores. VR_IR_ = Valence ratings of the independent raters (self-CIs); CS = Computational scores (self-CIs).

As expected, the correlation between the computational scores and the independent valence ratings evaluated was strongly significant (*r* = 0.80, *p* < 0.001). In addition, the computational scores were positively correlated with all variables related to self-image: self-esteem, *r* = 0.25, *p* < 0.01, explicit self-evaluation, *r* = 0.38, *p* < 0.001, and extraversion, *r* = 0.37, *p* < 0.001 (see Table 2). For better understanding of results of computational scores, we presented an imagery outcome in Figure 4. We separately averaged the self-CIs in high (+1 *SD*) and in low (−1 *SD*) groups of computational scores and of independent valence ratings (see Figure 4; two faces: female and male; two conditions: Experiment 2a and Experiment 2b). Taken together, these findings imply that the computational scoring method may be used to measure the valence of self-CIs more efficiently.

## 5. General discussion

The RC technique is a data-driven method that can provide a new perspective on self-image. We demonstrated that a mental representation of self is associated with not only self-reported variables related to self-image but also with social evaluation. More importantly, as we expected, the valence ratings of self-CIs evaluated by independent raters predicted the expert ratings on psychological adjustment, after controlling for the effects of self-reported self-esteem and extraversion. Also, despite the significant relationship between the independent valence ratings of self-CIs and facial appearance, only the independent valence ratings of self-CIs, but not the facial appearance, were significantly correlated with expert ratings. In addition, we provide evidence that the computational scoring method can supplement the independent rating process. The computational scores of self-CIs were closely related to the valence ratings of self-CIs by independent raters. Also, the computational scores were positively correlated with variables relevant to self-image and social evaluation.

This study is the first, to the best of our knowledge, to address the relationship between self-image visualized by means of the RC method and social evaluation by incorporating expert evaluations. Our findings are consistent with previous research suggesting that people with positive self-views tend to be perceived more favorably by others than those with negative self-views (Taylor and Brown, 1988; Taylor et al., 2003; Zeigler-Hill et al., 2013). Our results extend these findings by employing the RC method to show the significant association between self-image and social evaluation. Particularly, we confirmed the validity of self-CIs by using the evaluations of psychological adjustment by three clinical psychologists, which are generally deemed to be more credible than are those by untrained raters. More importantly, the self-CIs provided independent and incremental information to self-reported variables in predicting social evaluation. This implies that the RC method has an important advantage, in that it can provide additional information about self-image by making the ineffable explicit as a visual form (Mangini and Biederman, 2004; Moon et al., 2020).

One possible explanation for the information that self-CIs add to self-reports in predicting social evaluations could lie in the implicit nature of self-CIs. That is, self-CIs can reflect implicit attitudes toward self. For example, Dotsch et al. (2008) showed that individuals’ mental representations of racial faces generated by the RC method were associated with their level of implicit prejudice toward Moroccans, a highly stigmatized out-group in the Netherlands, measured by an Implicit Association Test (IAT). Paulhus (1984) also pointed out that when participants perform self-reports, their responses may be affected by self-deception and impression management, which are closely related to social desirability. However, in a RC task, participants can freely adopt any dimensions (e.g., emotional impressions or facial features) to make judgments about facial resemblance. Moreover, participants can be unaware of the criteria they adopt, because the RC approach allows them to make instantaneous and instinctive choices when selecting facial stimuli that more resemble their faces (see Brinkman et al., 2017, for review). In addition, previous studies have suggested that mental representations visualized through the RC method are less affected by certain response patterns or social desirability than are self-reported measures (e.g., Moon et al., 2020; Pauzé et al., 2021). Likewise, our findings showed that social desirability was not significantly related to self-CIs but was to self-image relevant variables, such as explicit self-evaluation and self-esteem. Thus, relying solely on self-reports to understand self-image may hinder a thorough comprehension of self-image. Taken together, our findings suggest that employing both self-reports and the RC method can lead to a better understanding of the link between self-image and social evaluation.

Some may raise an alternative explanation that the relationship between self-CIs and social evaluations is merely the effect of facial appearance, because participants with more attractive facial features can generate more attractive self-representations and can be evaluated more favorably by others in social situations (i.e., halo effect; Dion et al., 1972). However, our findings can rule out the aforementioned explanation. Although we concede that visual mental representations inevitably reflect facial appearance to some degree, we found that the valence ratings of self-CIs of participants were not explained solely by those of their facial appearance. Also, social evaluation was only associated with the self-CIs but not facial attractiveness. These results reveal that the visual proxies of self-image are not just a reflection of facial appearance but are a multifaceted composite colored by psychological factors (Maister et al., 2021).

Additionally, we present the first evidence that the computational scoring method can be valid and useful in assessing the valence of self-CIs across Experiment 2a and 2b. Prior studies have employed independent raters to evaluate participants’ self-CIs on valence (Moon et al., 2020; Steiner et al., 2021) and personality traits (Maister et al., 2021). However, incorporating newly recruited independent raters each time to evaluate self-CIs can cause inefficiency by necessitating substantial time and effort. Moreover, individuals’ self-CIs may be too noisy and unclear for independent raters to detect the inter-individual differences in the valence of self-CIs (Imhoff et al., 2013). Our findings show that the computational scoring method that we proposed can measure the valence of self-CIs objectively and efficiently without recruiting additional independent raters. We expect that this method can be extended to other areas of interest, such as competence and dominance.

### 5.1. Limitations and future directions

There are a few limitations of this study that need to be addressed in future research. First, we used only expert ratings to examine whether participants’ self-CIs were associated with how they were perceived by other people. However, the amount of information that experts can grasp about participants by means of a brief self-introductory video may be limited (Zeigler-Hill et al., 2013). Previous studies employing acquaintance evaluation have shown that the more information acquaintances know about participants, the more reliable and accurate their reports are (Paulhus and Bruce, 1992; Vazire, 2010). Thus, future studies can be designed to examine the relationship between participants’ self-CIs and social evaluations by close others (e.g., friends, romantic partners, and family members).

Second, another limitation in our paradigm was that we could not be sure whether participants selected the one from two facial stimuli that looked more like themselves or selected the stimuli that gave more positive impressions. However, we did not intend to rule out the possibility of incorporating impressions when making their choices. Rather, we expected the participants to make visual mental representations that inevitably incorporated factors like affective impression while selecting the one from two facial stimuli that looked more like themselves. Further, because participants were able to freely adopt criteria without *a priori* assumptions, we believed that resulting self-CIs can provide incremental information to participants’ facial appearance. Nevertheless, incorporation of an experimental method to systemically distinguish standards that participants employ while carrying out the RC task can lead to further understanding of the visual mental representation (Brinkman et al., 2019). In addition, future works can explore neural activations in brain regions related to self-other discrimination (e.g., the medial prefrontal cortex and the right temporo-parietal junction; D'Argembeau et al., 2007; Zeugin et al., 2020) to investigate specific neural mechanisms while participants perform the RC task to generate their self-CIs.

Finally, participants in this study mainly consisted of young adults and were limited to Asians, which means that the external validity of our results is quite restricted. Therefore, future studies need to be done on participants of various age groups to generalize the current findings, and need to examine whether our findings are applicable to participants of various ethnic groups.

## 6. Conclusion

We extend the validity of self-CIs by demonstrating that the significant relationship between self-image and social evaluation can be captured by means of the RC method. More importantly, we reveal that the self-CIs add information to self-reports and facial appearance in predicting social evaluation. Additionally, we propose that the computational scoring method complements the independent rating process in measuring the valence of self-CIs. Our findings suggest that self-CIs are a valid and useful tool to comprehend the relationship between self-image and social evaluation.

## Data availability statement

The datasets presented in this study can be found in online repositories. The names of the repository/repositories and accession number(s) can be found below: Open Science Framework (OSF): https://osf.io/rstwv/.

## Ethics statement

The studies involving human participants were reviewed and approved by the Institutional Review Board at Korea University (KUIRB-2019-0220-03 and KUIRB-2019-0221-01). The patients/participants provided their written informed consent to participate in this study.

## Author contributions

JK: conceptualization, methodology, investigation, software, visualization, formal analysis, validation, data curation, writing – original draft, writing – review and editing, project administration. KM: conceptualization, methodology, software, visualization, data curation, writing – original draft, writing – review and editing. SK: conceptualization, methodology, data curation, writing – original draft, writing – review and editing. HK: conceptualization, writing – review and editing, supervision. YK: conceptualization, methodology, writing – review and editing, supervision, project administration. All authors contributed to the article and approved the submitted version.

## Conflict of interest

The authors declare that the research was conducted in the absence of any commercial or financial relationships that could be construed as a potential conflict of interest.

## Publisher’s note

All claims expressed in this article are solely those of the authors and do not necessarily represent those of their affiliated organizations, or those of the publisher, the editors and the reviewers. Any product that may be evaluated in this article, or claim that may be made by its manufacturer, is not guaranteed or endorsed by the publisher.

## Footnotes

^1^Participants also completed the remaining 50 items on the HEXACO-60 scale. The scale assesses Honesty-Humility (H), Emotionality (E), Agreeableness (A), Conscientiousness (C), Openness to Experience (O), and Extraversion (E). Consistent with Moon et al. (2020), the other five dimensions were not significantly associated with the independent valence ratings of self-CIs or the computational scores of self-CIs in Experiment 1 and 2b, |*r*|s < 0.17, *p* = ns.

^2^ https://osf.io/rstwv/

^3^Participants also evaluated the valence of their self-CIs with seven items. We decided to only use the valence ratings of self-CIs by independent raters because the independent ratings of self-CIs were comparable to the valence ratings of self-CIs by the participants (see Supplementary Table S1 for details).

^4^ pavlovia.org

^5^We also asked the experts to evaluate the participants in the video with the same seven items for rating the valence of self-CIs. The results were generally consistent with the five items for psychological adjustment but less salient. Therefore, we did not include the valence ratings evaluated by experts in further analysis (see Supplementary Table S2 for details).

^6^ https://osf.io/rstwv/
