# Supplementary material for: The relationship between mental representations of self and social evaluation: Examining the validity and usefulness of visual proxies of self-image
Source: Front Psychol. 2023 Jan 12;13:937905. doi: 10.3389/fpsyg.2022.937905 (PMC9878293; doi:10.3389/fpsyg.2022.937905)
Supplement: Supplementary file 1 [file Table_1.DOCX]

Supplementary Material

# The valence ratings of self-CIs evaluated by the RC sample

Participants (RC sample), who performed the RC task to generate their self-CIs, also evaluated the valence of their self-CIs. As shown as Supplementary Table S1, the valence ratings of self-CIs rated by the RC sample were positively correlated with self-esteem (Experiment 1: *r* = .23, *p* < .05; Experiment 2b: *r* = .23, *p* < .01), explicit self-evaluation (Experiment 1: *r* = .25, *p* < .01; Experiment 2b: *r* = .33, *p* < .001), extraversion (Experiment 1: *r* = .24, *p* < .01; Experiment 2b: *r* = .37, *p* < .001), the valence ratings of self-CIs evaluated by independent raters (Experiment 1: *r* = .46, *p* < .001; Experiment 2b: *r* = .52, *p* < .001), and the computational scores of self-CIs (Experiment 2a: *r* = .43, *p* < .001; Experiment 2b: *r* = .46, *p* < .001). Also, the valence ratings of self-CIs rated by the RC sample were negatively correlated with anxiety (Experiment 1: *r* = -.22, *p* < .05; Experiment 2b: *r* = -.19, *p* < .05), and were not correlated with depression (Experiment 1: *r* = -.17, *p* = .069; Experiment 2b: *r* = -.11, *p* = .231) or social desirability (Experiment 1: *r* = .08, *p* = .398; Experiment 2b: *r* = .13, *p* = .153). However, unlike the valence ratings of self-CIs by independent raters, the RC sample’s valence ratings of self-CIs were not significantly associated with the valence ratings of facial appearance (Experiment 1: *r* = .08, *p* = .387), or expert ratings on psychological adjustment (Experiment 1: *r* = .16, *p* = .090) and on valence (Experiment 1: *r* = .06, *p* = .506).

**Supplementary Table S1.**

*Correlations between the valence ratings of self-CIs evaluated by the RC sample and other study variables*

|  | Experiment 1 and 2a | |  | Experiment 2b | |
| --- | --- | --- | --- | --- | --- |
|  | VR_RC_ | |  | VR_RC_ | |
| 1. VR_IR_ | .46 | ^***^ |  | .52 | ^***^ |
| 2. CS | .43 | ^***^ |  | .46 | ^***^ |
| 3. Self-esteem | .23 | ^*^ |  | .23 | ^**^ |
| 4. Explicit self-evaluation | .25 | ^**^ |  | .33 | ^***^ |
| 5. Extraversion | .24 | ^**^ |  | .37 | ^***^ |
| 6. Depression | -.17 |  |  | -.11 |  |
| 7. Anxiety | -.22 | ^*^ |  | -.19 | ^*^ |
| 8. Social desirability | .08 |  |  | .13 |  |
| 9. VR_FA_ | .08 |  |  | --- | |
| 10. Expert ratings |  |  |  |  |  |
| Psychological adjustment | .16 |  |  | --- | |
| Valence | .06 |  |  | --- | |
| *M* | 5.54 | |  | 5.41 | |
| *SD* | 1.15 | |  | 1.21 | |

*Note*. *N* = 118 / 127 (Experiment 1 and 2a / Experiment 2b). VR_IR_ = Valence Ratings of the Independent Raters (self-CIs); VR_RC_ = Valence Ratings of the Reverse Correlation sample (self-CIs); CS = Computational Scores (self-CIs).

^*^ *p* < .05, ^**^ *p* < .01, ^***^ *p* < .001.

# Expert ratings on the dimension of valence

In Experiment 1, the valence evaluated by experts were significantly associated with the valence ratings of self-CIs by independent raters (*r* = .20, *p* < .05). Among self-reported variables related to self-image, extraversion showed a positive correlation with expert ratings (*r* = .23, *p* < .05). In Experiment 2a, the valence evaluated by experts were positively correlated with the computational scores of self-CIs (*r* = .28, *p* < .01). Given that extraversion was positively correlated with valence evaluated by experts, we performed two multiple linear regression analyses predicting expert ratings on valence controlling for the effect of extraversion. In one model (Experiment 1), independent variables included the valence ratings of self-CIs rated by independent raters and extraversion; in another model (Experiment 2a), independent variables included the computational scores of self-CIs and extraversion. As shown in Table S2, the relationship between the valence ratings self-CIs evaluated by independent raters and expert ratings was no longer significant after controlling for the effect of extraversion. On the contrary, the effect of the computational scores of self-CIs on expert ratings remained significant after controlling for the effect of extraversion.

**Supplementary Table S2.**

*Results of the multiple regression analyses predicting the valence rated by experts*

| Predictors | Unstandardized Coefficients | |  | Standardized Coefficients | |  | *t*(115) | *R*^2^ |
| --- | --- | --- | --- | --- | --- | --- | --- | --- |
|  | *B* | *SE* |  | β | 95% CI |  |  |  |
| Model 1 |  | |  |  | |  |  | |
| VR_IR_ | .12 | .08 |  | .14 | [-0.04, 0.33] |  | 1.53 | .07 |
| Extraversion | .02 | .01 |  | .19^*^ | [0.00, 0.38] |  | 2.01 |  |
|  |  | |  |  | |  |  | |
| Model 2 |  | |  |  | |  |  | |
| CS | 4.57 | 1.92 |  | .22^*^ | [0.04, 0.41] |  | 2.38 | .10 |
| Extraversion | .02 | .01 |  | .16 | [-0.03, 0.34] |  | 1.68 |  |

*Note*. *N* = 118. *F*(2, 115) = 4.47^*^ (Model 1), 6.24^**^ (Model 2). VR_IR_ = Valence Ratings of the Independent Raters (self-CIs); CS = Computational Scores (self-CIs).

^*^ *p* < .05, ^**^ *p* < .01.
